# Tracking Fungal Community Responses to Maize Plants by DNA- and RNA-Based Pyrosequencing

**DOI:** 10.1371/journal.pone.0069973

**Published:** 2013-07-18

**Authors:** Eiko E. Kuramae, Erik Verbruggen, Remy Hillekens, Mattias de Hollander, Wilfred F. M. Röling, Marcel G. A. van der Heijden, George A. Kowalchuk

**Affiliations:** 1 Department of Microbial Ecology, Netherlands Institute of Ecology (NIOO-KNAW), Wageningen, The Netherlands; 2 Department of Ecological Science, VU University Amsterdam, Amsterdam, The Netherlands; 3 Department of Molecular Cell Physiology, VU University Amsterdam, Amsterdam, The Netherlands; 4 Research Station ART, Agroscope Reckenholz Tänikon, Zurich, Switzerland; 5 Department of Biology, Utrecht University, Utrecht, The Netherlands; University of Tartu, Estonia

## Abstract

We assessed soil fungal diversity and community structure at two sampling times (t1 = 47 days and t2 = 104 days of plant age) in pots associated with four maize cultivars, including two genetically modified (GM) cultivars by high-throughput pyrosequencing of the 18S rRNA gene using DNA and RNA templates. We detected no significant differences in soil fungal diversity and community structure associated with different plant cultivars. However, DNA-based analyses yielded lower fungal OTU richness as compared to RNA-based analyses. Clear differences in fungal community structure were also observed in relation to sampling time and the nucleic acid pool targeted (DNA versus RNA). The most abundant soil fungi, as recovered by DNA-based methods, did not necessary represent the most “active” fungi (as recovered via RNA). Interestingly, RNA-derived community compositions at t1 were highly similar to DNA-derived communities at t2, based on presence/absence measures of OTUs. We recovered large proportions of fungal sequences belonging to arbuscular mycorrhizal fungi and *Basidiomycota*, especially at the RNA level, suggesting that these important and potentially beneficial fungi are not affected by the plant cultivars nor by GM traits (Bt toxin production). Our results suggest that even though DNA- and RNA-derived soil fungal communities can be very different at a given time, RNA composition may have a predictive power of fungal community development through time.

## Introduction

Numerous soil processes are primarily, and some even exclusively, carried out by soil fungi. Two of the most important processes are the facilitation of nutrient uptake and degradation of crop remains [1], [2]. The fungi responsible for these key soil functions are in direct contact with plants and plant materials. Therefore, they may be especially vulnerable to alterations in e.g. plant defenses and carbohydrate composition and availability [3], [4], as generated by Genetic Modification (GM) or other plant variety specific differences.

Recently, methodological advances have allowed enhanced screening of soil microbial communities, revealing high diversity and temporal turnover based on sequencing of 18S rRNA gene variants [5]. In addition, high-throughput sequencing approaches, such as 454 pyrosequencing, have greatly expanded the power of such nucleic acid-based assessments of complex fungal communities [6], [7]. Nuclear SSU rRNA genes have proven to be useful markers for assessing fungal community structure across a range of habitats, including soils [7], [8]. However, it has been demonstrated that non-active or dormant fungi may persist in sampled DNA pools, potentially masking the dynamics of the more active constituents of fungal communities [9]–[11]. As an alternative approach, environmental 18S rRNA can be targeted by RT-PCR amplification from soil RNA extracts [12]–[14] in order to focus on the active components of fungal communities [12], [15], [16]. The combination of DNA- and RNA- based assessments of fungal communities is therefore thought to provide a more complete picture of fungal community dynamics [16]. However, there is currently no consensus on whether DNA, RNA or both should be targeted to obtain the most meaningful assessment, and how communities obtained by analysing either nucleic acid type relate to each other.

In this study, we examined the potential impact of four maize plant cultivars, consisting of two genetically modified cultivars (GM) and two near-isogenic non-GM cultivars, on soil-borne fungal communities in a pot-based experiment. GM plants were incorporated into the study to address concerns that some transgenic crops may adversely affect soil processes, including important groups of beneficial soil fungi. To date, most studies on this topic have been focused on the effects of Bt (*Bacillus thuringiensis* toxin coding gene introduced in maize) crops on arbuscular mycorrhizal fungi (AMF). Some of these studies have reported no consistently significant impacts on AMF [17]–[21], although others have detected significant negative effects of Bt plants on AMF [22], [23].

To provide an in-depth and comprehensive account of fungal communities, we targeted 18S rRNA genes and reverse-transcribed 18S rRNA at two sampling times (t1 = 47 and t2 = 104 days of plant age) by 454 pyrosequencing, and the relative impacts of plant cultivar, plant age and nucleic acid pool were assessed. A previous study using the same system [21] focused on solely AMF (average of 12% total sequence reads) and related their diversity in pot experiments to natural AMF community variation in the field. In the current study, we assess the full fungal community to examine the potential impact of GM-cultivars on soil fungal diversity and community structure, and specifically determine whether DNA and RNA generally reflect similar community dynamics and how these two different nucleic acid pools relate to each other.

## Materials and Methods

### Plant Cultivars and Experimental Setup

The four different maize (*Zea mays* L.) cultivars used in this study consisted of “Monumental MON810” (GM event MON810) and “DK 3421YG” (GM event MON810) and two near isogenic non-GM cultivars of these lines (“Monumental” and “DKC 3420”, respectively). For brevity, different cultivars are abbreviated by letters, Monumental = M, DKC = D, and GM cultivars are indicated with “GM” (i.e. M - GM, and D – GM). Both GM cultivars had been transformed to express the *CryIAb* gene (an insecticidal endotoxin produced by *Bacillus thuringiensis* that is active against, among others, the European corn borer *Ostrinia nubilalis*).

Intact pot-size soil cores from an organically managed agricultural field were transferred to pots in order to maintain natural stratification and integrity of fungi inhabiting the soil. No specific permits were required for this field sampling. This field is not a protected area and does not involve endangered or protected species. Soil cores were collected randomly from within a homogeneous 10×10 m plot in September 2009. The standing crop was a grass-clover mixture (*Trifolium pratense* L. and *Lolium perenne* L.), which had been sown after maize in the autumn of 2007 and mown twice a year. Soil chemical properties were pH (CaCl_2_-extractable) = 5.8, P (CaCl_2_-extractable) = 5.1 mg kg^−1^, N = 1.36 g kg^−1^, OM = 1%. In each pot (containing approximately 6 kg of soil, diameter = 20 cm, height = 18 cm), one of four different maize (*Zea mays* L.) cultivars was grown.

The experimental design was 2 GMs×2 non-GMs×3 replicate pots×2 sampling times×2 types of nucleic acids. Two seeds were sown into each pot on October 1^st^ 2009, and pots kept in a greenhouse (16/8 hours light/dark). One seedling was kept per pot after two weeks. Hoagland solution (½ strength P; 250 ml per pot) was applied twice during the first month of plant growth. On November 24^th^, after 47 days (t1) of plant growth, soil samples were taken in the following way: one core (diameter 1 cm) per pot was taken and the part originating from 5–11 cm depth was immediately transferred to dry ice and subsequently stored at −80°C. Cores were taken 5–6 cm from the edge, which was approximately halfway between the edge of the pot and the stem of the plant. On January 20^th^ 2010, after 104 days (t2) of growth, samples were again taken as above, but the position of cores was shifted 45° in relationship to the first core to minimize potential disturbances from the first sampling event. At the end of the experiment (plant age 130 days; at full maturity of the ears), total above- and belowground plant biomass was harvested.

### Nucleic Acid Extraction and cDNA Preparation

RNA and DNA were simultaneous extracted from two grams of fresh bulk soil (soil without plant roots) per sample using the RNA PowerSoil kit and the DNA Elution accessory kit (MO BIO Laboratories inc., Carlsbad, CA, USA). The DNA from total RNA samples was digested by DNase I (RNase-free DNase set Qiagen 79254) according to manufacturer recommendations. The total RNA was measured with a ND-1000 spectrophotometer (Nanodrop Technology, Wilmington, DE, USA), and the quality of the total RNA was checked with Experion (BioRad). The cDNA was synthesised from the total RNA using random hexamer primers and the superscript double-stranded cDNA synthesis kit (Invitrogen, Life Technologies) exactly as described in Verbruggen et al. 2012.

### Nucleic Acid Amplification and Sequencing of 18S rRNA Gene Fragments

DNA and cDNA templates derived from each of the soil pots at t1 = 47 days and t2 = 104 days of plant age were utilized for nucleic acid amplification and subsequent 454 pyrosequencing. In these procedures, a fungal-specific primer set was used, consisting of the FR1 primer [24] and a modified version of the FF390 primer ([24]; 5′-CGWTAACGAACGAGACCT-3′), to allow for inclusion of the *Glomeromycota* (Arbuscular Mycorrhizal Fungi). Thermocycling conditions were: denaturing at 94°C for 30 s (after initial denaturation of 4 min. initial annealing temperature was 55°C (1 min.), and every two cycles the annealing temperature was lowered by 2°C until 47°C was reached, which was the annealing temperature used for the final twenty cycles (thus, 29 PCR cycles in total). Extension conditions were 68°C for 2 min. for all cycles. Reactions contained about 25 ng of DNA or cDNA template added to a standard PCR mix. Four PCR reactions of 25ul each per biological replicate of a sample were carried out. Primers contained 12 different multiplex tags for sample identification were sequenced in 1/8 lane plate of Roche 454 automated sequencer and GS FLX system using titanium chemistry (454 Life Sciences, Branford, CT, USA). Sequence analysis was performed using QIIME 1.2.1 scripts [25] incorporated into the Galaxy interface [26]. We generally used DNA and cDNA (RNA) of three biological replicates per sample; however, eight samples [M (DNA-t1), M (DNA-t2), M (RNA-t1, M (RNA-t2), D (DNA-t1), D (DNA-t2), D (RNA-t1), D (RNA-t2)] had two replicates due to technical issues. All sequencing reads were checked for containing the right forward and reverse MID-tags and assigned to samples accordingly. Then, barcodes and tags were removed, and sequences were denoised using Denoiser 0.91 [27] and clustered at 97% similarity using the UCLUST 1.2.21 algorithm [28]. The resulting “operational taxonomic units” (OTUs) were assigned to eukaryote families through BLAST searches against the QIIME-compatible version of the Silva 104 release [29]. Singleton OTUs were removed for statistical analyses. The OTU table was rarefied to 906 reads using “single rarefaction” QIIME script since this number was the lowest number of reads in any single sample after singleton removal. This rarefied OTU table was used for all subsequent statistical analyses. Shannon (diversity), evenness and richness (Chao1) were calculated in the PAST program [30], and the percentage of coverage was calculated by Good’s method [31]. Good’s coverage estimator is a method of estimating what percent of the total species, in this case OTUs, is represented in a sample, which estimator equation gives a good idea of how their limited sampling relates to the entire sampled community. Two-way PERMANOVAs were separately performed for testing GM (Bt versus non-Bt maize) and cultivar (each of the two parental cultivars) effects and for DNA and RNA in response to sampling time using the Vegan package [32] in R (The R Foundation for Statistical Computing). Jaccard’s similarity was calculated based on OTU presence and absence in PAST program [30].

## Results

### Diversity of Fungal Communities

After denoising, chimera detection and removal of non-fungal reads (34% DNA; 24% RNA), a total of 60,845 reads were used for analyses. The average length of reads of fungal 18S rRNA was 375 bp. The numbers of reads of fungal DNA-based ranged from 1,025–6,970 while from RNA-based ranged from 2,448–9,000. Although we sought to mix PCR products in equal amounts prior to sequencing, product amounts were quantified using a spectrophotometer. Such measures may not be entirely accurate, and may have caused undesired variations in template amounts and subsequently sequence numbers.

In order to compare samples, we discarded singletons and rarefied the OTU table to the lowest numbers of reads obtained for any single sample. Doing this, we ended up with 906 reads per sample, yielding between 76 and 97 OTUs per sample, and OTU coverage ranged from 92 to 97% as determined by Good’s coverage estimator (Table 1). OTU number comparisons between non-GM versus GM plants, different sampling times (t1 = 47 days; t2 = 104 days) or nucleic acid type were not significantly different (Table 1).

**Table 1:** Estimators of sequences diversity, evenness, richness and coverage given by environmental DNA and RNA in soils with two maize cultivars (M = Monumental; D = DKC3420) and their respective genetically modified lines (M-GM = event MON810; D-GM = DKC3421YG) at different sampling times (t1 = 47 days; t2 = 104 days) after sowing.

	Number of OTU^(1)(2)^	Singletons(2)	Shannon (diversity)(2)	Evenness(2)	Chao-1 (richness)(2)	Good’s coverage estimator(2)
**Cultivar**						
M (DNA-t1)	80(3)±11a	36±28	3.30±0.04a	0.35±0.06a	110±44a	94±5
M (DNA-t2)	85±2a	24±6	3.43±0.37a	0.38±0.13a	98±2a	96±1
M-GM (DNA-t1)	94±9a	40±1	3.21±0.32a	0.27±0.06a	126±2a	93±0
M-GM (DNA-t2)	76±10a	17±19	3.48±0.27a	0.44±0.13a	89±28a	97±3
M (RNA-t1)	95±19a	37±14	3.32±0.08a	0.30±0.05a	126±35a	94±2
M (RNA-t2)	86±10a	38±15	3.24±0a	0.30±0.03a	133±43a	94±2
M-GM (RNA-t1)	78±5a	36±6	2.79±0.18a	0.21±0.05a	127±20a	94±1
M-GM (RNA-t2)	94±15a	48±13	2.85±0.36a	0.19±0.03a	155±48a	92±2
D (DNA-t1)	87±8a	27±2	3.45±0.21a	0.37±0.04a	103±8a	96±0
D (DNA-t2)	80±12a	21±6	3.22±0.22a	0.32±0.02a	90±3a	97±1
D (RNA-t1)	78±13a	41±9	2.68±0.44a	0.19±0.05a	137±24a	93±2
D (RNA-t2)	89±6a	38±7	3.20±0.26a	0.28±0.05a	131±24a	94±1
D-GM (DNA-t1)	80±15a	29±5	3.13±0.45a	0.30±0.08a	111±13a	95±1
D-GM (DNA-t2)	85±13a	35±15	3.07±0.11a	0.26±0.03a	120±41a	94±2
D-GM (RNA-t1)	93±12a	50±7	2.75±0.21a	0.17±0.02a	181±3a	92±1
D-GM (RNA-t2)	97±14a	49±18	2.83±0.37a	0.19±0.08a	175±61a	92±3
*P value*	ns	ns	ns	ns	ns	
**Non-GM/GM**						
Non-GM	85±6a	33±8	3.23±0.24a	0.31±0.06a	116±18a	95±1
GM	87±8a	38±11	3.01±0.25a	0.25±0.09a	135±32a	94±2
*P value*	ns	ns	ns	ns	ns	
**Sampling time**						
t1	85±7a	37±7	3.08±0.29a	0.27±0.07a	128±24	94±1
t2	86±7a	34±12	3.16±0.23a	0.29±0.09a	124±31	94±2
*P value*	Ns	ns	ns	ns	ns	
**Nucleic acid type**						
DNA	82±6a	28a ±7	3.28±0.16a	0.33±0.06	105±12	95a ±1
RNA	88±7a	42b ±6	2.96±0.25a	0.23±0.06	146±22	93b ±1
*P value*	Ns	*	ns	ns	*	

(1) Operational Taxonomic Unit.

(2) The values are mean of replicates (n = 2–3).

(3) Values with the same letters were not significantly (ns) different (*P*<0.05); *P<0.05Significant comparisons at p<0.05.

Fungal diversity (Shannon), evenness and richness (Chao-1) indices per plant cultivar were not different (Table 1). Similar results were also observed in soils with different non-GM and GM plants and across the sampling times, t1 and t2. However, the Chao-1 richness index between nucleic acid types, DNA versus RNA, was significantly different. The DNA-based (t1, t2) richness (Chao-1) was lower than that derived from RNA templates (t1, t2) index (Table 1).

### Comparison of Fungal Communities

Differences were detected in soil fungal community composition associated with the different plant genetic backgrounds examined in the experiment. There is a significant effect of plant cultivar in DNA-based analysis for sampling time t2 and no significant effect of GM for both DNA and RNA-based analysis at the same sampling time t2 (Table 2). There is a significant difference between DNA and RNA-microbial community based analysis (Table 2).

**Table 2:** Results of two-way PERMANOVA testing the effect of “GM” (Bt *vs.* non-Bt maize) and “cultivar” (each of the two types of parental cultivars).

Nucleic acid	Time		Df	F	R^2^	P
DNA	t1	GM	1	0.77	0.09	0.72
		Cultivar	1	0.89	0.10	0.55
		GM*Cultivar	1	1.01	0.12	0.41
		Residuals	6	0.69		
	t2	GM	1	1.55	0.14	0.08
		Cultivar	1	**1.87**	**0.17**	**0.02**
		GM* Cultivar	1	1.41	0.13	0.13
		Residuals	6	0.55		
RNA	t1	GM	1	1.53	0.16	0.12
		Cultivar	1	1.25	0.13	0.24
		GM* Cultivar	1	0.84	0.09	0.58
		Residuals	6	0.62		
	t2	GM	1	2.09	0.20	0.05
		Cultivar	1	1.65	0.16	0.11
		GM* Cultivar	1	0.59	0.06	0.83
		Residuals	6	0.58		
			Df	F	R^2^	P
DNA		Time	1	**6.32**	**0.26**	**<0.001**
		Residuals	18	0.74		
RNA		Time	1	**3.30**	**0.16**	**0.002**
		Residuals	18	0.84		

Tests were performed separately for each nucleic acid type at each time point (t1 = 47 days and t2 = 105 days of plant age). Significant values are indicated in bold. The bottom part of the table represents separate PERMANOVA’s for DNA and RNA in response to the factor “sampling time”.

**Figure 1:**
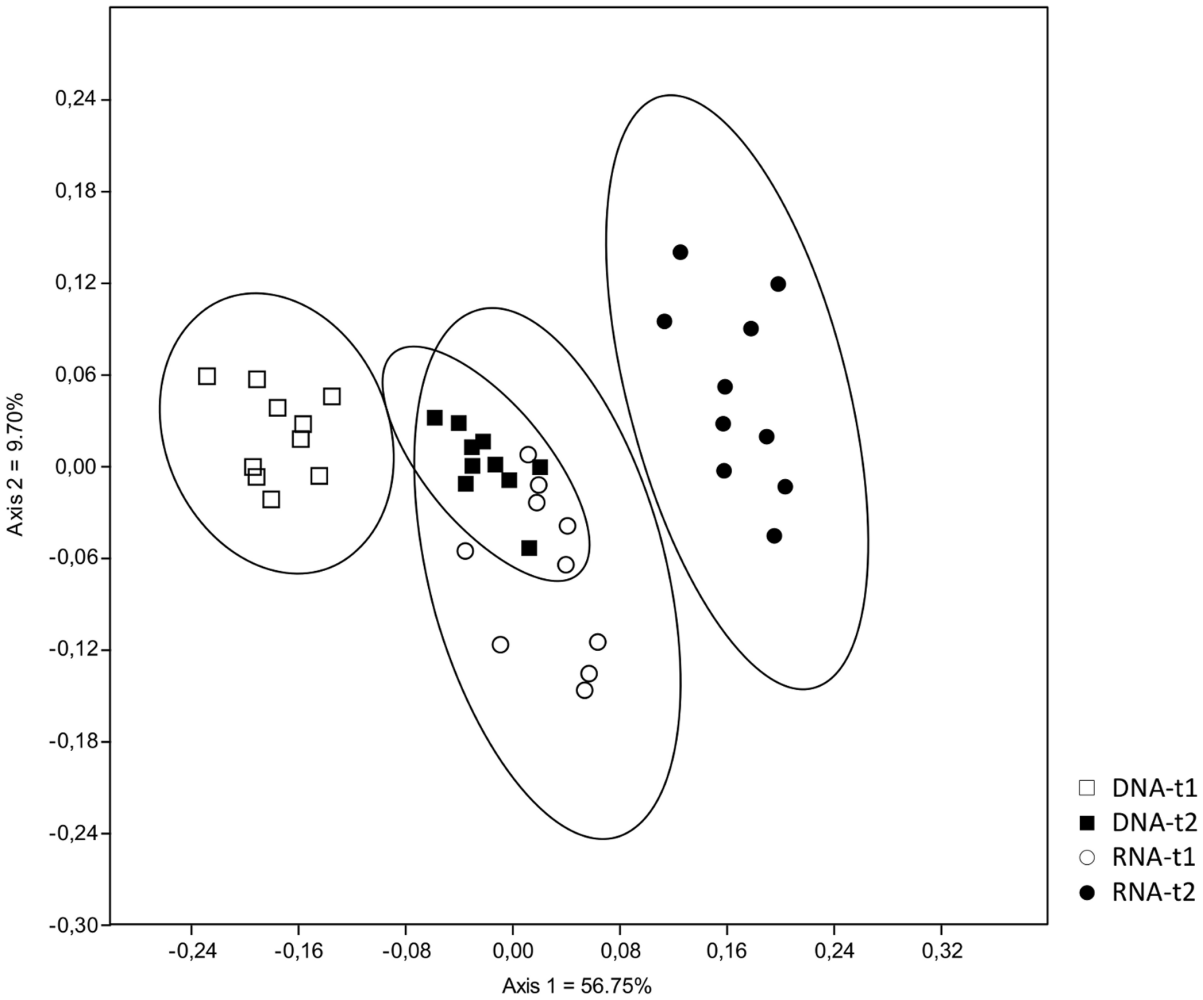
Non-metric multidimensional scaling (NMDS) analysis of presence and absence of fungal OTUs based on Jaccard-index of similarity with 95% confidence intervals shared between sampling times (t1 = 47 days; t2 = 104 days) and nucleic acid type (DNA, RNA).

**Figure 2:**
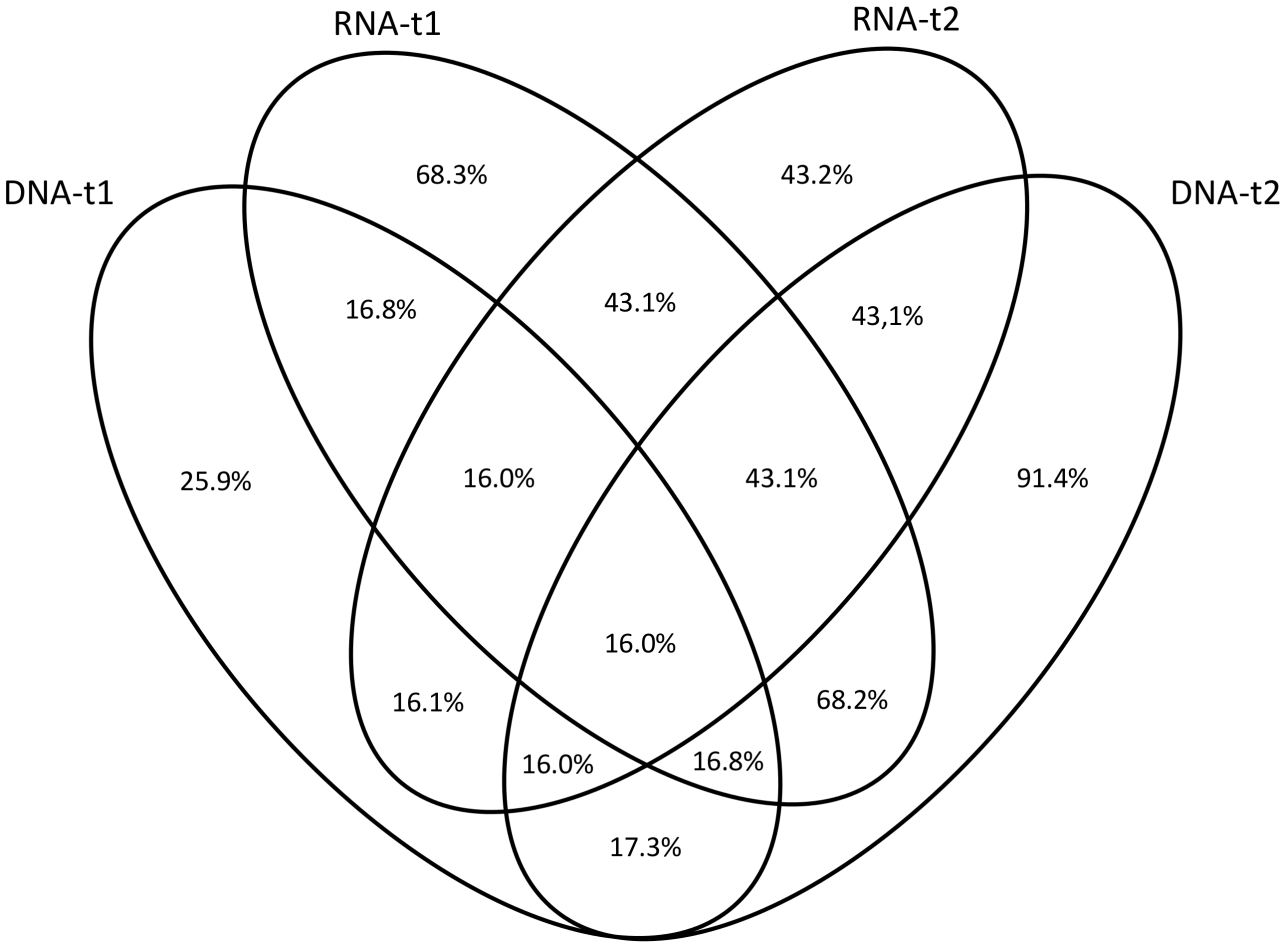
Percentage of fungal OTUs shared between nucleic acid type (DNA; RNA) in different sampling times (t1 = 47 days; t2 = 104 days).

**Figure 3:**
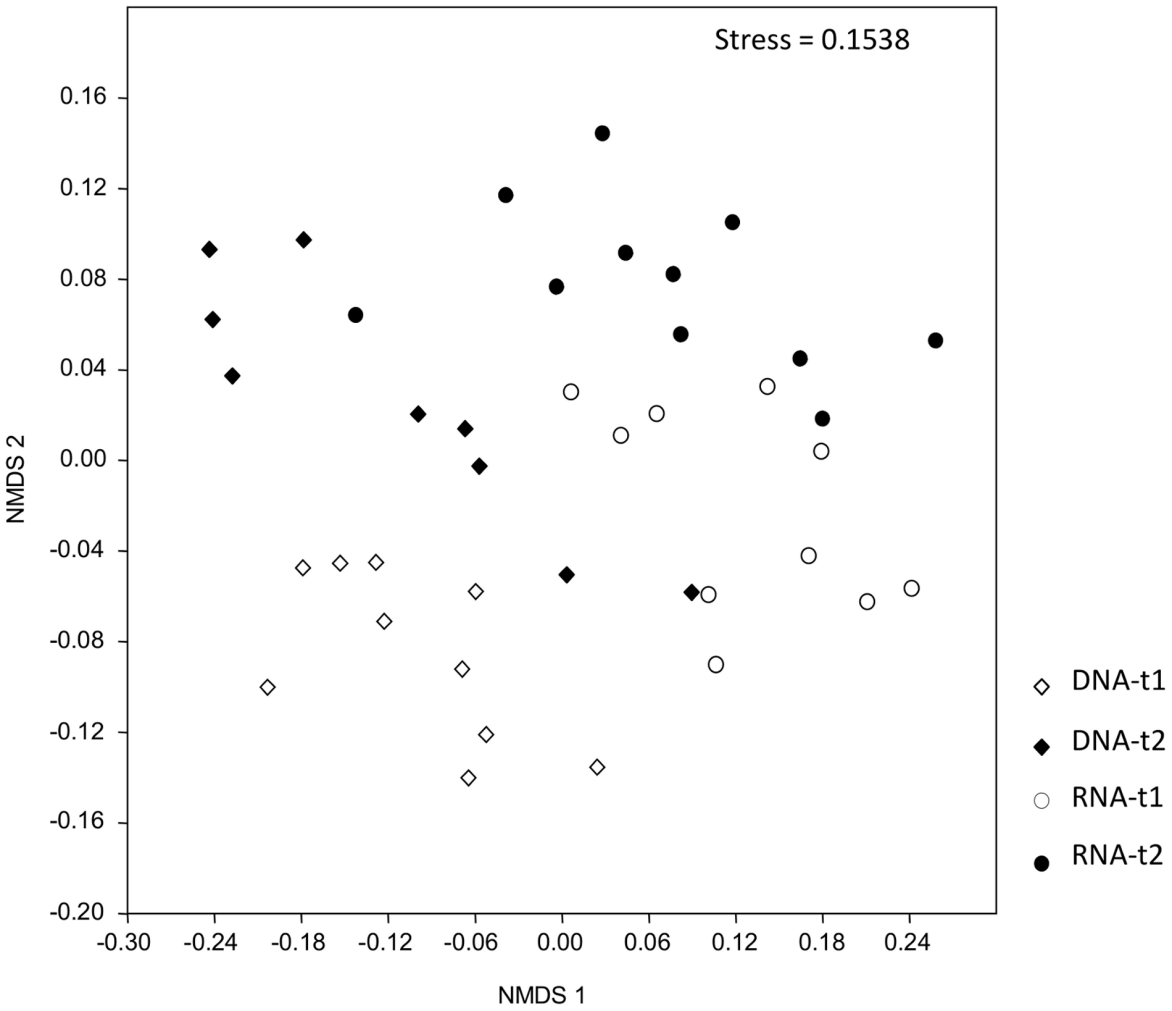
NMDS analysis based on relative abundance of fungal OTUs based on environmental DNA and RNA both at sampling times t1-47 days and t2-104 days of plant age.

When OTU occurrence between sampling times was compared using a presence/absence measure of similarity (Jaccard), a very clear separation of treatments was observed. All nucleic acid type/time combinations formed distinct clusters, with the exception of the RNA-based OTUs at sampling time t1 and the DNA-derived OTUs at sampling time t2, which grouped very close together and were overlapping (Figure 1). Moreover, these two groups shared a large number of OTUs (Figure 2), despite the separation by nucleic acid type and time observed based on relative abundance measures (Figure 3). This indicates that taxa relative read number within treatment is rather dynamic, but that taxa occurrence at a given sampling time represented by either DNA or RNA is relatively more predictable.

**Figure 4:**
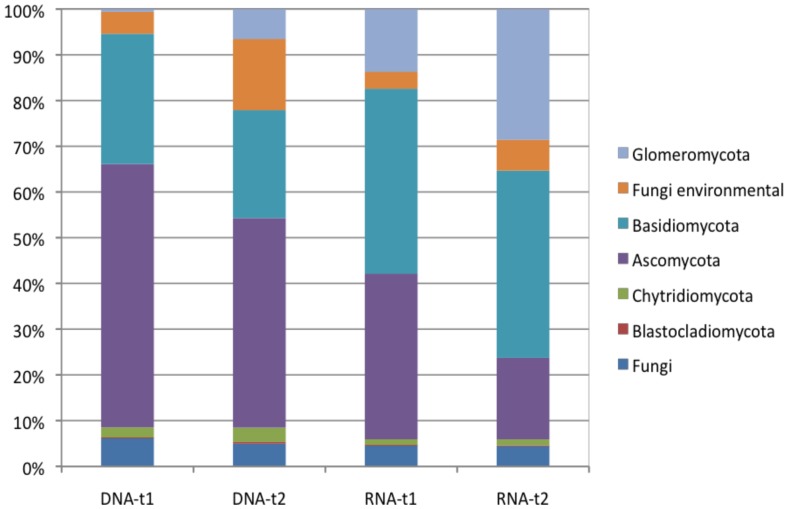
Relative abundance of different fungal phyla recovered from environmental DNA and RNA in soils with two maize cultivars (M = Monumental; D = DKC 3420) and their respective genetically modified lines (M-GM = event MON810; D-GM = DKC 3421YG) at two different sampling times (t1 = 47 days; t2 = 104 days) of plant age.

There were different patterns in fungal phyla obtained by DNA-t1 and DNA-t2-based analysis, and by RNA-t1 and RNA-t2-based analysis. In the DNA-based fungal community, *Ascomycota* was the most abundant phylum independent of the sampling time, while in the RNA-based fungal community, *Basidiomycota* was the most abundant phylum for both time points. The *Glomeromycota* were more abundant in the RNA-derived datasets, and increased in relative abundance with plant age (t2) (Figure 4).

The relative abundance of members within different fungal phyla from the DNA- and RNA-based analyses were also significant different. The relative abundances of “environmental fungi” (sequences only known from direct sequencing on nucleic acids isolated from environmental samples), environmental *Chytridiomycota*, *Ascomycota* (environmental, *Sordariomycetes*, *Saccharomycetales*) and *Glomeromycota* (*Glomerales*) were significantly higher in the DNA-derived dataset, while the relative abundance of *Basidiomycota* (*Agaricales*, environmental) was higher in the RNA dataset (Table 3).

**Table 3:** Relative abundance (%) of soil fungal groups based on pyrosequencing analysis of environmental DNA and RNA in soils with two maize cultivars and their respective genetically modified lines at different sampling times (t1 = 47 days; t2 = 104 days) of plant age.

	DNA-t1	DNA-t2	RNA-t1	RNA-t2	DNA	RNA	DNA *vs.* RNA	DNA-t1 *vs.* DNA-t2	RNA-t1 *vs.* RNA-t2
Fungi environmental	4.8±0.04	15.6±0.59	3.7±0.08	7.1±0.60	10.2±0.58	5.40±0.45	**	***	ns
Chytridiomycota (environmental)	1.2±0.06	1.7±0.05	0.8±0.03	0.8±0.03	1.5±0.05	0.80±0.03	**	ns	ns
Ascomycota (environmental)	3.5±0.01	1.8±0.09	1.1±0.02	0.7±0.03	2.65±0.11	0.90±0.03	***	**	ns
Ascomycota (mitosporic)	1.8±0.02	1.1±0.04	1.4±0.05	1.3±0.05	1.45±0.03	1.35±0.05	ns	**	ns
Ascomycota (Dothideomycetes)	2.0±0.03	1.2±0.06	1.2±0.06	2.1±0.06	1.60±0.05	1.65±0.07	ns	**	**
Ascomycota (Sordariomycetes)	37.7±0.89	30.2±0.48	27.1±0.79	13.2±0.79	33.95±0.72	20.15±0.71	***	ns	**
Ascomycota (Saccharomycetales)	9.8±0.35	8.3±0.32	1.0±0.04	0.5±0.04	9.05±0.32	0.75±0.05	***	ns	ns
Basidiomycota (Agaricales)	1.3±0.07	1.4±0.05	2.2±0.04	1.7±0.04	1.35±0.06	1.95±0.04	**	ns	**
Basidiomycota (environmental)	14.5±0.44	16.4±0.26	31.4±0.86	29.6±0.86	15.45±0.33	30.50±1.05	***	ns	ns
Glomeromycota (Glomerales)	0.5±0.02	4.9±0.13	13.1±0.32	25.0±0.32	2.70±0.15	19.05±0.73	***	***	ns
Glomeromycota (Paraglomerales)	0.5±0.01	4.9±0.08	13.1±0.07	25.0±0.07	0.85±0.07	2.50±0.13	ns	***	ns

Numbers are mean of replicates.

Non-Parametric MANOVA (NPMANOVA) test between samples based on Bray-Curtis distance measure. Significance levels: ns: *P*>0.05;

** *P*<0.005;

*** *P*<0.0005.

Relative abundances lower than 0.1% is not shown.

In the DNA-based analysis, the relative abundances of members of “environmental fungi”, and *Glomeromycota* (*Glomerales*, *Paraglomerales*) significantly increased with plant age, while members of the *Ascomycota* (environmental, mitosporic, *Dothideomycetes*) decreased (Table 3). For RNA-derived sequences, the relative abundance of *Ascomycota* (*Dothideomycetes*) and *Glomeromycota* (*Glomerales*, *Paraglomerales*) significantly increased with plant age, while *Ascomycota* (*Sordariomycetes*) and *Basidiomycota* (*Agaricales*) significantly decreased (Table 3).

At the same time point of sampling (t1 or t2), the relative abundances of fungal groups based on DNA and RNA analyses were not the same. At sampling time t1, members of the *Ascomycota* (environmental *Ascomycota*, *Dothideomycetes*), *Basidiomycota* (*Agaricales*) and *Glomeromycota* (*Glomerales*) were significantly different between DNA and RNA-based analysis (Table 3). Also, at time point t2, members of environmental fungi, *Ascomycota* (environmental, *Dothideomycetes*, *Sordariomycetes*) and *Glomeromycota* (*Diversisporales*, *Glomerales*) were significantly different between DNA and RNA-fungal community based analysis (Table 3).

## Discussion

Despite having to restrict our analysis to relatively small numbers of sequence reads per sample, we still recovered 92 to 97% of the total OTU-level fungal diversity in the soil samples studies as determined by Good’s coverage estimator. The numbers of OTUs obtained in this study were rather low compared to other soils i.e. forest soils [6], [7]. However, caution must be used in comparing diversity estimates using different markers. In the current study, we targeted a rather conserved region of the 18S rRNA gene, which only provides a taxonomic discrimination to approximately the order level. The use of a more variable marker, such as the Internal Transcribed Spacer (ITS) regions [33], which can discriminate to the subspecies level, would no doubt yield larger OTU numbers than we recovered.

Under the greenhouse conditions used in this study, no differences in soil fungal diversity (Shannon), evenness and richness (Chao-1) indices could be detected with respect to the GM nature of the plant. However, there was a difference in fungal richness depending on the type of nucleic acid (DNA or RNA) and the time point of soil sampling. The fungal community assessed by DNA-based analysis was richer than fungi community assessed by RNA-based analysis.

The fungal community structure was also not affected by non-GM and GM plants. This result is in agreement with previous studies that have examined the colonization and community structure of mycorrhizal fungi in response to GM lines of maize [17], [21], soybean [34], cotton [18] and tobacco [35]. Our inability to detect shifts in total fungal community structure is in contrast with the observations of Tan et al. [34], who observed distinct cultivar effects on *Glomus-*like fungi, as determined by PCR-DGGE community profiling. Although these authors did observe differences between GM plants and their nearly isogenic parental lines, these differences were not greater that those observed between different non-GM cultivars. These previous studies have zoomed in specifically on AMF, yet relatively few studies have examined complete fungal community responses to GM plants [36]–[39], however none of these studies applied next generation sequencing approach as addressed in the current study. Our results indicate that the observed lack of a GM-related effects in our experiment was not caused by a low resolution of our assessment, but rather by weak effects of plant cultivar compared to other community-structuring processes because i) fungal communities of 47 and 104 days of plant growth were clearly distinct, and plant age was the most important explanatory factor of fungal community composition, and ii) we observed clear differences in the fungal community structures as recovered from DNA versus RNA templates. Regarding this last observation, these differences do not seem to be caused by a fundamental difference between the taxa recovered as DNA compared to RNA (in that some are very active but low in abundance or vice-versa), but rather indicate a dynamic system where taxa measured by RNA give an indication of taxa found as DNA later in the season (*i.e.* the t1 RNA-based community appears to foreshadow the t2 DNA-based community). It should be noted, however, that we did not examine any samples in the intervening time between the two sampling points, and additional observations and time points would be necessary to confirm the time-scale and generality of this relationship between RNA versus DNA template pools.

Although DNA and RNA clearly give rise to potentially complementary windows of observation, it should be kept in mind that ribosome number (*i.e.* 18S rRNA content) may be differentially correlated with activity for different species [40], and both DNA and RNA measurements have specific biases (DNA; e.g. copy number differences between species, RNA; different ribosome numbers per cell). Thus, while RNA-based analyses may provide additional insight into the generally active populations in environmental samples, one cannot extract precise activity estimates based upon such approaches.

In conclusion, soil fungal community proved to be dynamic with changes over time across nucleic acid pools, but not revealing any detectable impact of maize cultivar or the GM nature of the plant.

### Accession Numbers

Nucleotide sequences were deposited in GenBank-SRA under the Study Accession No. ERP002065 for agriculture soil samples.
